# Supplementary material for: Control of Gelation and Properties of Reversible Diels–Alder Networks: Design of a Self-Healing Network
Source: Polymers (Basel). 2019 May 28;11(6):930. doi: 10.3390/polym11060930 (PMC6630651; doi:10.3390/polym11060930)
Supplement: Supplementary file 1 [file polymers-11-00930-s001.pdf]

Supplementary Material

Control of gelation and properties of reversible Diels-Alder networks. Design of a self-healing network

Beata Strachota, Adama Morand, Jiří Dybal, Libor Matějka

[1] Institute of Macromolecular Chemistry, Academy of Sciences of the Czech Republic, Heyrovsky Sq. 2, 162 06 Prague 6, Czech Republic; beata@imc.cas.cz (B.S); dybal@imc.cas.cz (J.D); matejka@imc.cas.cz (L.M)
[2] Sigma Clermont, Campus des Cezeaux, 63178 Aubiere, France; Adama.Morand@sigma-clermont.fr
* Correspondence: matejka@imc.cas.cz

Characterization of the tetrafunctional furan monomer F4D2000
FTIR-ATR, cm^{-1}: 3440 (OH str), 1513, 1504 (C=C str), 1223 (COH bend), 1149 (COC str), 921(epoxy), 750, 740 (C-H out of plane bend). ^{1}H NMR (CDCl3, 300 MHz), δ/ppm: = 4.50, 6.31, 6.68, 7.35.
The extent of the reaction was 95%.

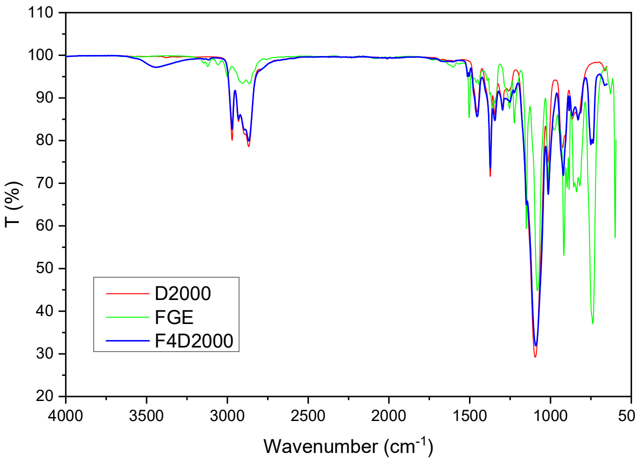

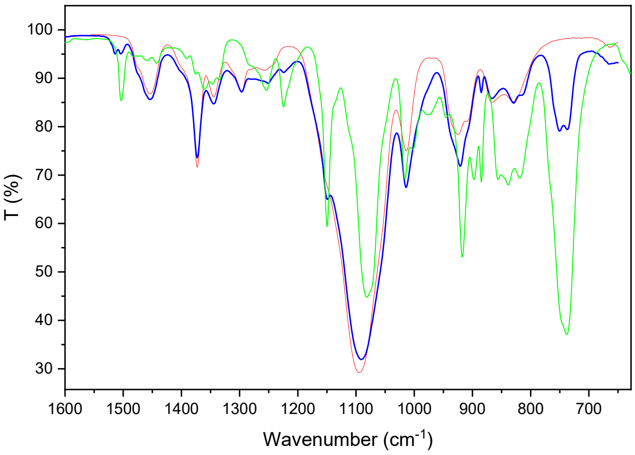

Figure S1. FTIR spectrum of F4D2000, Jeffamine D2000 and FGE. Detail of the spectrum.

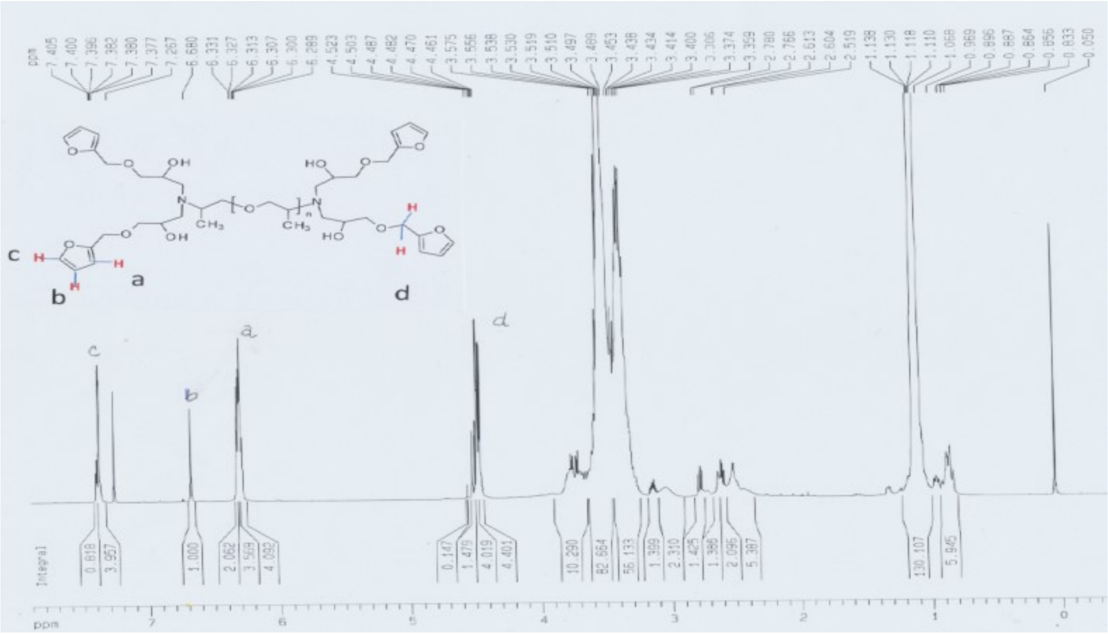

Figure S2. ^{1}H NMR spectrum of F4D2000.

Characterization of F6T3000

FTIR-ATR, cm^{-1}: 3440 (OH str), 1513, 1504 (C=C str), 1223 (COH bend), 1149 (COC str), 921(epoxy), 750, 740 (C-H out of plane bend). ^{1}H NMR (CDCl3, 300 MHz), δ/ppm: = 4.50, 6.31, 6.68, 7.38.
The extent of the reaction was 95%.

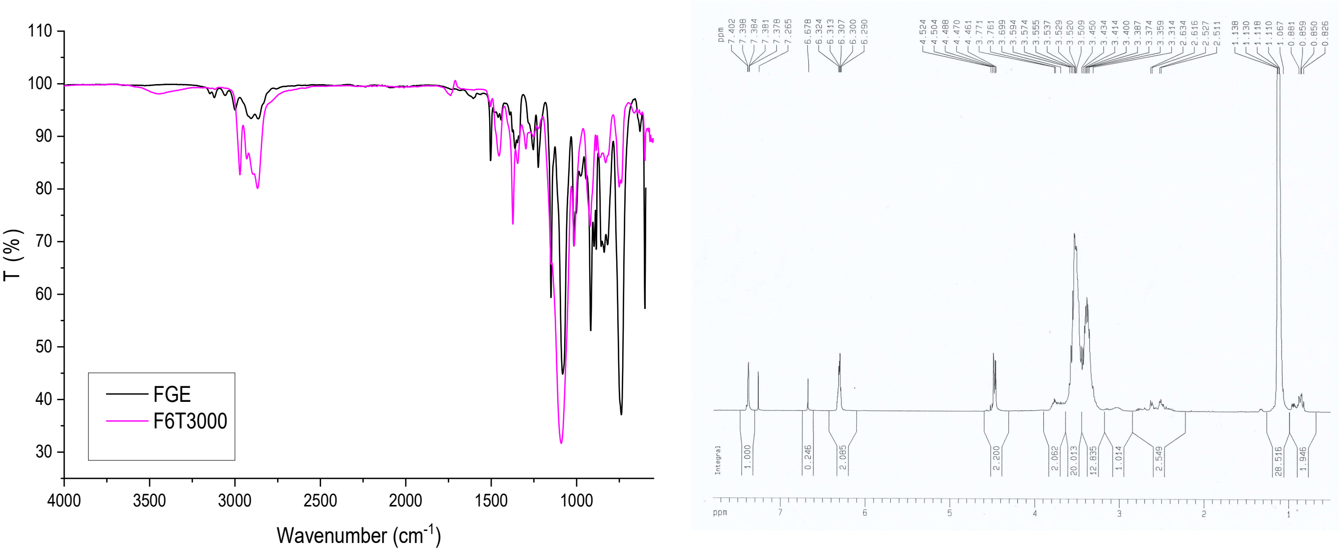

Figure S3. FTIR and ^{1}H NMR spectra of F6T3000

Characterization of F3FAFGE

FTIR-ATR cm^{-1}: 3440 (OH str), 3099 (=CH str), 2948 (asCH_2), 1134 (C–N str).
^{1}H NMR ($CDCl_3$, 300 MHz), δ/ppm: = 3.51, 4.46, 6.31. 7.35
The extent of the reaction was 95 %.

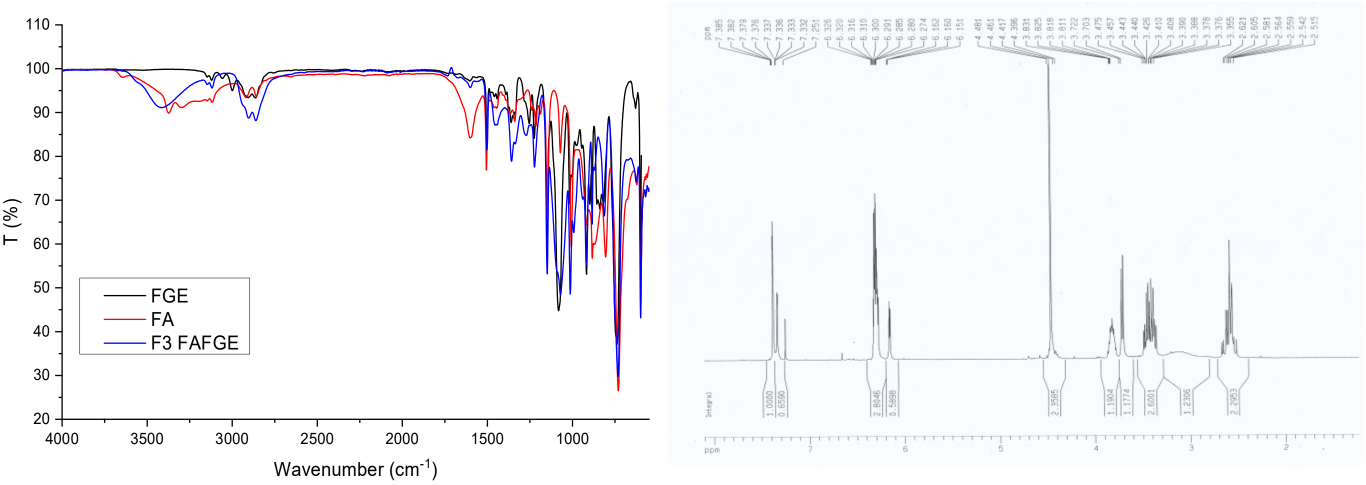

Figure S4. FTIR and ^{1}H NMR spectra of F3FAFGE

Characterization of N,N′-hexamethylenebismaleimide (HBMI)

FTIR-ATR (cm^{-1}): 3088 (=CH str), 2908 (CH_2), 2857 (CH_2 sym str), 1759 (C=O str), 1454 (CH_2 deform), 1372 (CH_2 wag), 1129 (C-N-C str), 946 (CH bend), 838 (=CH bend out of plane), 786 (CH_2 rock), 695 (ring breathing). ^{1}H NMR ($CDCl_3$, 300 MHz), δ/ppm: 1.28 (s, 2H), 1.55 (s, 2H), 3.5 (t, 2H), 6.67(s, 2H).
The extent of the reaction was 98 %.

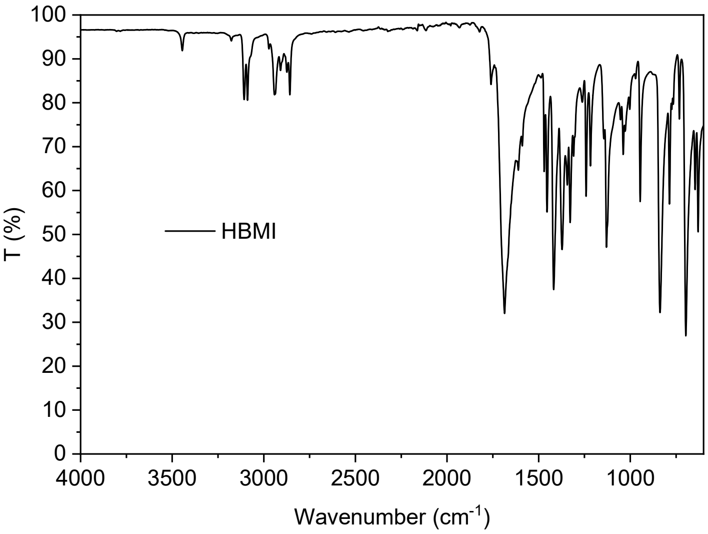

Figure S5. FTIR spectrum of HBMI

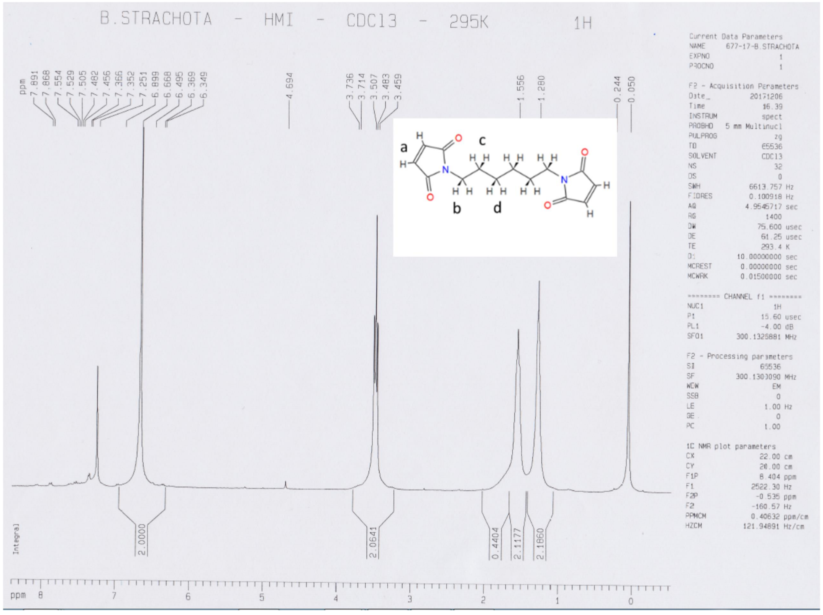

Figure S6. ^{1}H NMR spectrum of HBMI

Characterization of FMA: furan–maleic anhydride DA adduct (3,6-oxygen bridge-1,2,3,6-four hydrogen phthalic anhydride)

<u>FTIR-ATR</u>, cm^{-1}: 3123 (=CH str), 3002 (CH str), 1856 (C=O str in phase), 1776 (C=O str out of plane), 1309 (C-H bend), 1211 (C-O-C str), 1145 (C-H bend), 1084 (C-O, C-C str), 901 (C-H bend out of plane), 733 (C-H bend out of phase).<u>^{1}H NMR</u> (DMSO-d_6, 300 MHz), δ/ppm: = 3.3 (s, 2H), 5.34 (s, 2H), 6.56 (s, 2H).

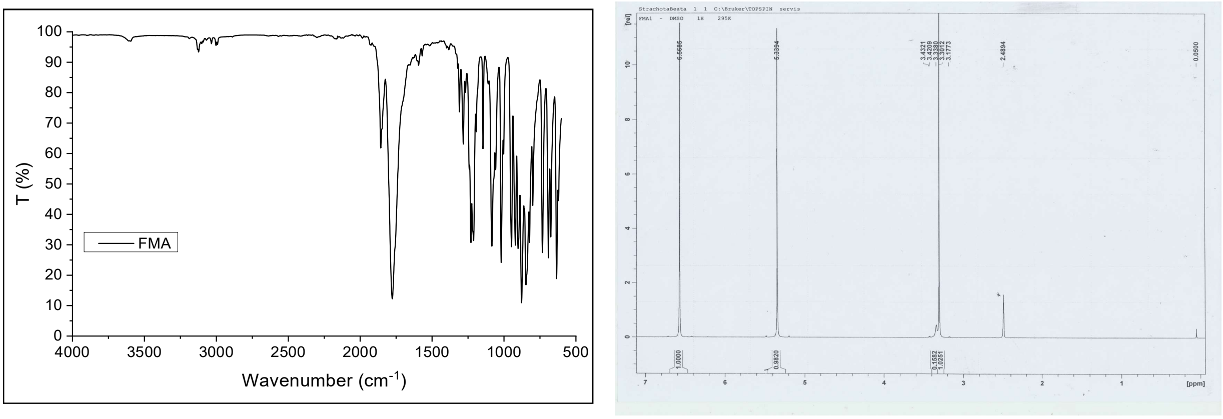

Figure S7. FTIR and ^{1}H NMR spectra of FMA

Characterization of the Tris(2-maleimidoethyl)amine (TMIEA)

FTIR-ATR cm^{-1}: 3099 (=CH str), 2948 (asCH_2), 2832 (sCH_2), 1770 and 1692 (C=O str), 1134 (C–N str).

^{1}H NMR ($CDCl_3$, 300 MHz), δ/ppm: = 2.7 (t, 2H), 3.51 (t, 2H), 6.67 (s, 2H).

The extent of the reaction was 99 %.

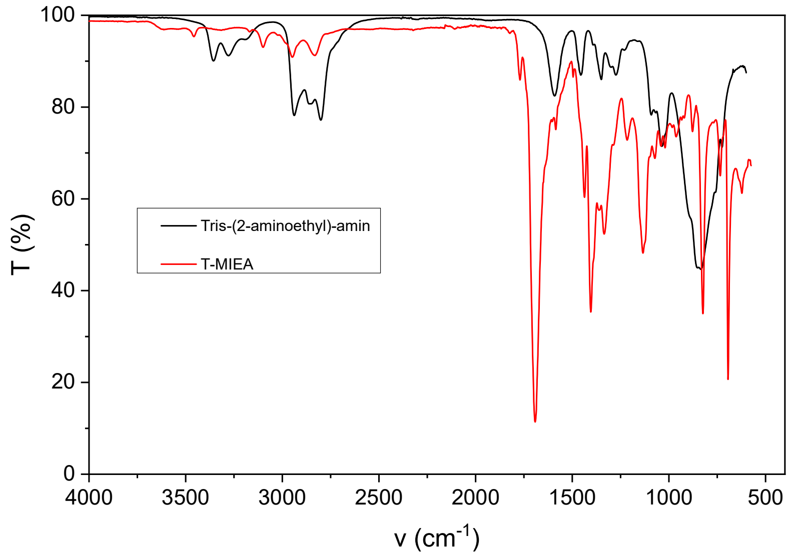

Figure S8. FTIR spectrum of TMIEA and Tris(2-aminoethyl)amine

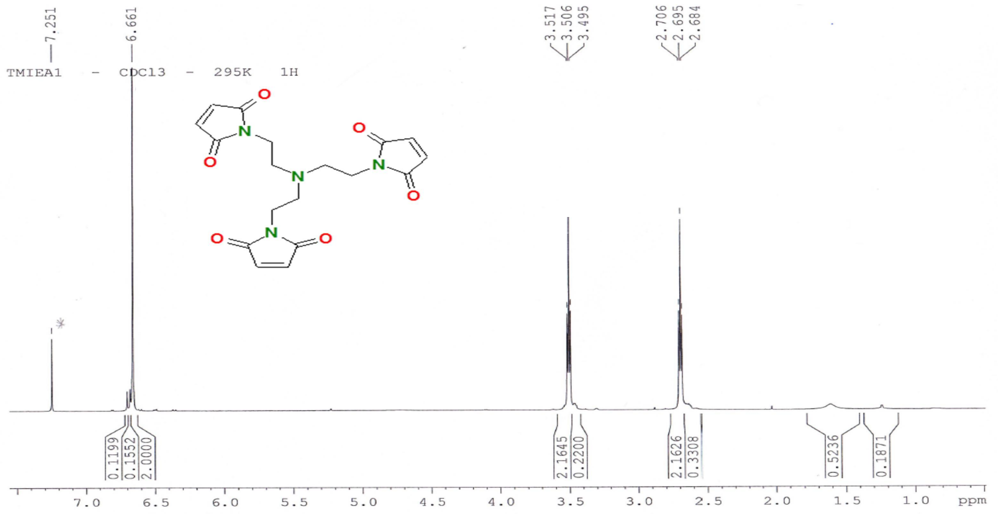

Figure S9. ^{1}H NMR spectrum of TMIEA

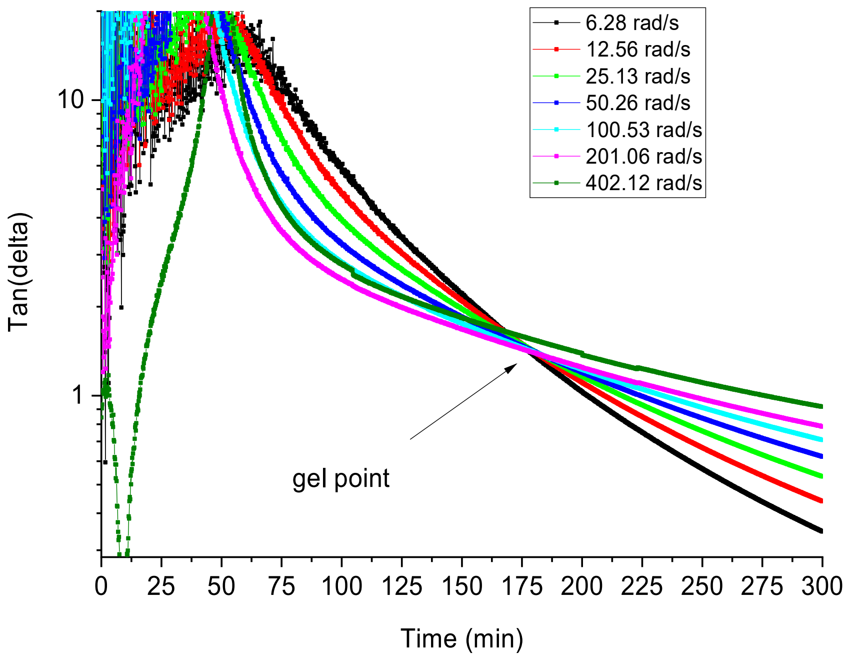

Figure S10. Determination of the gel point using the multifrequency sweep during the formation of the network F4D2000-PPO3BMI at 65°C. Angular frequencies, 6.28 – 402.12 rad/s.

Phase separation in reversible networks

The F4D2000-HBMI network is heterogeneous in the early reaction state as shown in Fig. S5a. At cooling after decrosslinking the network is formed and in the early stage two phases are obvious in Fig. S5a. The corresponding peaks at the tan δ curve are assigned to the PPO phase of furan and to the formed bismaleimid containing interphase at ~ -10 °C. During the reaction, the mixture is homogenized, the interphase disappeared and the cured network is homogeneous with the *Tg* peak at ~ -20 °C (Figure S5b).

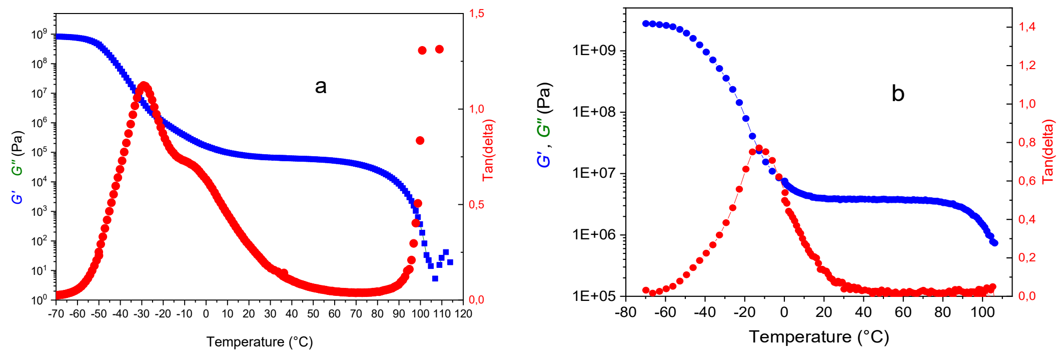

Figure S11. The storage modulus and loss factor tan δ of the network F4D2000-HBMI as a function of temperature. a) the early reaction state, b) the cured network

Table S1. Rate constants of the DA and rDA reactions in the F4D2000-bismaleimide mixture with different bismaleimides in solution and in bulk, respctively

bismaleimides	*T*, °C	50		90	
		solution	bulk	solution	bulk
	**k_{DA}** $lmol^{-1}min^{-1}$				
PPO3BMI		1.3E-2	7.8E-2	4.3E-2	11.3E-2
DPBMI		0.6E-2	7.4E-2	2.7 E-2	17.8E-2
HBMI		0.4E-2	21.8E-2	2.0 E-2	95 E-2
	**k_{rDA}** min-1				
PPO3BMI		1.5E-4	9.1E-4	1.6E-3	8.0E-3
DPBMI		0.9E-4	69.4E-4	3.4E-3	30 E-3
HBMI		0.2E-4	5.2E-4	1.4E-3	3.5E-3

Theory of branching processes

Theory of branching processes [TBP] [38] describes network formation and evolution of the branched structure during polymerization. The network formation proceeds by the reaction of f_F-functional furan with f_M-functional maleimide monomers. The TBP describes the system by distribution of structural units defined by the reaction state of the functional groups, i.e. number of reacted and unreacted functionalities. The distribution developing during the reaction is obtained by using a kinetic scheme. The structural units are combined at any moment of the reaction to form tree-like structures. The polymer characteristics evolving during network formation, such as molecular mass, fraction of the gel, concentration of elastically active network chains (EANC), etc. are calculated by using probability generating functions (pgf) describing the number of issuing bonds from a unit. The pgfs for a unit in a root are as follows:

$F_{0F}(z_M) = (1-\alpha_F + \alpha_F z_M)^{fF}$ for the furan

$F_{0M}(z_F) = (1-\alpha_M + \alpha_M z_F)^{fM}$ for the maleimide

$F_0 (z) = n_F F_{0F} (z_M) + n_M F_{0M} (z_F)$

α_M and α_F are conversions of the maleimide and furan groups, respectively. z is a dummy variable and subscripts of z indicate the direction of a bond, thus z_M and z_F indicate a bond from unit F (furan) to M (maleimide) and from M to F, respectively.

We calculated by using TBP the evolution of concentration of elastically active chains (EANC), crosslinking density (ν) and fraction of the sol (w_S) as a function of the reaction conversion α. For the stoichiometric composition of tetrafuran-bismaleimide (F4-M2) network is holds :

$$[EANC] = 3\alpha^2(1-v_M)^2 [1- (1- \alpha + \alpha v_M)]^2$$

$$\nu = 0.5 [EANC] / d(0.5M_F +0.5M_M)$$

$$w_S= m_F(1- \alpha + \alpha v_M)^4 + m_M (1- \alpha + \alpha v_F)^2$$

M_F, M_M and m_F, m_M are molecular weights and mass fractions of furan and maleimide monomers, respectively. v_F and v_M are the extinction probabilities calculated by iteration.
